# Adults’ spatial scaling of tactile maps: Insights from studying sighted, early and late blind individuals

**DOI:** 10.1371/journal.pone.0304008

**Published:** 2024-05-30

**Authors:** Magdalena Szubielska, Marta Szewczyk, Paweł Augustynowicz, Wojciech Kędziora, Wenke Möhring

**Affiliations:** 1 Faculty of Social Sciences, Institute of Psychology, The John Paul II Catholic University of Lublin, Poland; 2 Independent Researcher, Poland; 3 Faculty of Psychology, University of Basel, Basel, Switzerland; 4 Department of Educational and Health Psychology, University of Education Schwäbisch Gmünd, Germany; Universite de Rouen Normandie, FRANCE

## Abstract

The current study investigated spatial scaling of tactile maps among blind adults and blindfolded sighted controls. We were specifically interested in identifying spatial scaling strategies as well as effects of different scaling directions (up versus down) on participants’ performance. To this aim, we asked late blind participants (with visual memory, Experiment 1) and early blind participants (without visual memory, Experiment 2) as well as sighted blindfolded controls to encode a map including a target and to place a response disc at the same spot on an empty, constant-sized referent space. Maps had five different sizes resulting in five scaling factors (1:3, 1:2, 1:1, 2:1, 3:1), allowing to investigate different scaling directions (up and down) in a single, comprehensive design. Accuracy and speed of learning about the target location as well as responding served as dependent variables. We hypothesized that participants who can use visual mental representations (i.e., late blind and blindfolded sighted participants) may adopt mental transformation scaling strategies. However, our results did not support this hypothesis. At the same time, we predicted the usage of relative distance scaling strategies in early blind participants, which was supported by our findings. Moreover, our results suggested that tactile maps can be scaled as accurately and even faster by blind participants than by sighted participants. Furthermore, irrespective of the visual status, participants of each visual status group gravitated their responses towards the center of the space. Overall, it seems that a lack of visual imagery does not impair early blind adults’ spatial scaling ability but causes them to use a different strategy than sighted and late blind individuals.

## 1. Introduction

Studying people with blindness, and in particular people who are blind from birth offers a unique opportunity to investigate how visual experience may change human’s cognitive functioning. With respect to visual experience, previous research typically distinguished between congenitally blind or early blind people (i.e., individuals who cannot see from birth or early childhood and, consequently, cannot use visual representations) and late blind people (i.e., who lost sight later in life and have visual memories). Blind people were often tested with respect to their spatial cognition (for reviews, see [1, 2]). This body of research has typically indicated that early and congenitally blind people show comparable spatial performance as compared to sighted people and late blind people. It seems that they successfully compensate for their lack of vision and the impossibility of using visual mental representations by relying on different strategies. In general, early blind people tend to use verbal or kinesthetic strategies whereas sighted and late blind people tend to use visual-spatial strategies based on mental visualization [3–6].

### 1.1 The role of visual experience in mental imagery

So far, the role of visual experience in spatial cognition was investigated in research on mental synthesis [7], mental rotation [8–15], mental scanning [16, 17], spatial updating [9, 18–21], and other “active” spatial imagery tasks, such as mentally tracking a target’s pathway [22], folding a grid [4, 6] or zooming in or out the size of an object [23–27]. As spatial scaling can be considered as one type of imagery operation, it is likewise relevant to study the role of visual experience on this respective imagery process.

Mental imagery is closely connected to the visuo-spatial scratchpad in working memory (for a discussion, see [28]). More specifically, any transformation of a mental representation of an object or scene requires imagery processes. As postulated by Likova [28] (based on Baddeley’s [29] classic model of working memory), these transformations occur within a structure of working memory, namely the amodal spatial scratchpad. The idea of amodality of the spatial scratchpad seems rather uncommon to cognitive psychologists who often conduct studies among the sighted population in the visual domain. However, researchers testing blind participants or multisensory cognition in behavioral and neuroscience studies agree about the amodal (also termed modality-independent, supramodal, metamodal) character of human cortical organization and spatial representations (e.g., [2, 28, 30], for reviews, see [21, 31, 32]).

Because people who are blind from birth or early childhood cannot use mental visual representations, it is reasonable to expect some differences in mental imagery between early blind individuals versus sighted or late blind people. Iachini and Ruggiero [17] suggest that the difference in imagery processing between people with different visual status is quantitative in nature, given that they have found a classic mental scanning effect irrespective of visual experience. Their findings indicated that the linear function between the distance scanned on a mental map as well as the response times required for scanning was less evident albeit still significant in blind participants, especially in congenital blind participants.

Other researchers propose a qualitative difference because the mechanisms underlying imagery may differ between people with different visual experience. Metaphorically these different imagery mechanisms have been described as the “mind’s eye” [33] versus the “mind’s hand” [16, 34]. Researchers supporting the qualitative difference propose that imagery of early blind people cannot be “vision-like” as some researchers suggest (see e.g., [35]) due to the specific characteristics of the “mind’s hand”. Visual mental representations inherent in the “mind’s eye” seem to be holistic, high in resolution, and constructed by adopting an allocentric perspective [33]. In contrast, oppositional features characterize the “mind’s hand”. Based on a review of research on mental imagery in congenitally blind people, Szubielska [34] argues that the “mind’s hand” generates representations sequentially, and is not holistic in nature. The “mind’s hand” seems to construct representations successively similar to haptic perception. Perceiving through touch is time-consuming and takes place piece by piece. Among people deprived of any visual experience, these details can hardly be assembled into a coherent recognizable whole [36, 37]. Furthermore, this haptic task is challenging and requires cognitive effort, especially for more complex spatial stimuli [38, 39]. In support of this argumentation, imagery representations of early blind people indicate a person-related (egocentric) frame of reference [40] and seem to have a relatively low resolution [41, 42].

Summing up, regardless of the visual status, people may use mental imagery when performing spatial tasks. However, sighted and late blind individuals tend to use visual mental imagery that has both spatial and pictorial components (for a discussion, see [43]). In contrast, congenitally and early blind participants may apply mental imagery coherent with the “mind’s hand”, containing only spatial components.

### 1.2 Using mental maps in blind individuals

Blind people are able to form and use mental maps [44, 45]. Based on an interview survey with blind people, Hersh [46] found that participants used spatial imagery, and some also explicitly used the terms “mental maps”. Following their descriptions, they navigated efficiently when traveling on actual routes using these mental maps. In line with this finding, there is also empirical evidence that learning about a new environment is more effective when blind adults use tactile (i.e., embossed) maps to familiarize themselves with new routes. Using these maps, they navigated quicker and more correctly as compared to learning by direct experience and/or with verbal descriptions of a guide [47–49]. The beneficial effect of providing tactile information was even higher when tactile information was complemented with an audio description [50]. A recent study also showed that a touchscreen-based multimodal interface, called a vibro-audio map, was as effective for learning a new space as a tactile map [51]. Studies extended these findings even to children. Case studies have shown that 5-year-old [52] and 4-year-old [53] blind children can use simple tactile maps to guide their locomotion in real-world spaces and locate themselves. Further studies demonstrated that blind children can accurately locate themselves on a map and follow the route on the map that they take in a larger space [54] and tactile maps facilitated their performance in a large-scale space [55]. In a nutshell, these pieces of evidence suggest that blind people, including children, have some ability to efficiently use tactile maps.

One ability that is included in every map-reading task refers to spatial scaling. This ability to scale spatial information can be described as being able to relate spatial information in different-sized spaces [56], such as relating distance information provided in a map to the distance information in the referent space (i.e., the town). Several of the above-mentioned studies demonstrate that blind participants were able to scale spatial information up (e.g., when following a path in a large space based on a tactile map) and scale information down (e.g., when moving in a large space and asked to estimate the own location on a map).

Some studies investigating the usage of cognitive maps in sighted and blind adults focused on acquiring route and survey knowledge [57–59]. These studies showed that early and late blind people can form cognitive maps on the basis of route and survey descriptions [58, 59]. However, blind adults (irrespective of the onset of blindness and having visual memories) benefitted more than sighted adults from route descriptions when solving spatial problems [57, 58]. AAnother study examined blind adults and sighted controls in labyrinth tasks [60] and found comparable performance between these groups. A similar conclusion was made for children blind from birth [61] but see [12, 62].

Overall, these previous studies demonstrated several similarities albeit small differences in creating mental maps or reading tactile maps between blind and sighted individuals. However, none of these studies did systematically investigate spatial scaling. This lack of research is surprising as exploring this integral ability in map reading and understanding the underlying strategies used in spatial scaling are intriguing and may have practical implications for blind people’s independent mobility.

### 1.3 Spatial scaling strategies in the haptic domain

Spatial scaling is an important spatial ability and comes into play whenever we relate spatial information between a small- (or large-) scaled model and the referent it stands for (e.g., interpreting the large-scaled model of a cell and relating information to the real-life referent). Consequently, this ability is involved in many daily situations and particularly in education and professions. Thus, it is not surprising that this respective ability has been specified as an overarching theme for science, technology, engineering, and mathematics disciplines by the National Research Council of the United States [63].

A common approach to assess spatial scaling is to present participants with a map containing a target and ask them to remember this position and to indicate the same position in an empty referent space that differs in size from the map [64]. Typically, the time taken to perform the scaling task (i.e., response times), and errors serve as dependent variables. Scaling factor is systematically manipulated by using different ratios between the size of the map and the referent space. By varying the scaling factors, it is possible to identify underlying spatial scaling strategies.

Three strategies have been described in the previous literature as defined by differential patterns of response times and errors [65, 66]. In the absolute distance strategy, participants may encode spatial information provided in a map in an absolute way and match this information onto a given referent space. This strategy is especially error-prone with higher scaling factors, with errors increasing with higher scaling factor. At the same time, such an absolute strategy may not affect participants’ response times given that the mapping time should remain the same irrespective of the scaling factor. A second relative distance strategy points to the proportional encoding of spatial information. In this strategy, participants may encode relative distances (i.e., a target being one-fifth from the left landmark) and map this information on a referent space [67, 68]. This strategy would work irrespective of size differences between the map and the referent space. Therefore, participants’ errors and response times are expected to remain constant across different scaling factors. A third strategy refers to using mental transformation strategies. This strategy builds upon findings in classic mental-imagery research [33, 69–71]. Similar to the mental-imagery processes shown in this research, participants may encode spatial information in the map by constructing a mental image and mentally transforming this image to map it onto the referent space. In line with this research [33, 71], this mental scaling would take longer and be more error-prone with larger transformations, as indicated by higher response times and errors with larger scaling factors. Studies examining adults’ spatial scaling in the visual domain revealed evidence for mental transformation strategies [64, 66].

The majority of spatial scaling research has been conducted in the visual domain [56, 64–67, 72–78], with only few studies investigating spatial scaling in the haptic domain [79–85]. Importantly, so far, it remains poorly understood how blind individuals who rely permanently on haptic perception scale spatial information. Furthermore, as outlined above, blind individuals vary with respect to the visual experiences and ability to use visual imagery. Here, it may be the case that individuals with visual experiences (i.e., late blind individuals, [24]) use other scaling strategies than congenitally blind or early blind individuals without visual memory (cf. [18]).

This difference can be expected based on findings showing that congenitally blind individuals have often used an egocentric reference frame when performing spatial tasks. Such egocentric strategies are body-centered and depend on body movements [40]. By contrast, allocentric strategies do not use the own body as a reference but use relations between the presented objects instead. Late blind and sighted individuals were more likely than early and congenitally blind people to use an allocentric reference frame [18, 58, 86–91]. Therefore, it seems that visual experience is crucial for developing accurate allocentric representations [92]. It can be expected that in spatial scaling tasks, these egocentric strategies used by early blind individuals are less effective because these representations are less flexible and it is more difficult to adapt them to the changed scale [23, 24, 27]. By contrast, mental transformation strategies might be considered as allocentric strategies that are more flexible and include an adaptation to size changes inherent in the spatial scaling task. Researchers suggest that using mental transformation strategies in spatial scaling is associated with visualizing (haptic) stimuli [80, 83]. Thus, individuals deprived of any visual experiences in life may be inclined to use other spatial scaling strategies.

To our knowledge, there is only one study that examined how congenitally blind individuals scale spatial information [81]. This study investigated congenitally blind participants and blindfolded sighted controls who were matched on several demographic variables. Findings indicated that congenitally blind participants performed less accurately in the spatial scaling task than the blindfolded sighted controls. However, these higher errors occurred because congenitally blind individuals mixed up the left and right side of the space more often than blindfolded sighted individuals. When accounting for these reversal errors, spatial scaling performance did no longer differ between the groups. Another similarity between congenitally blind participants and sighted controls concerned the response strategies when locating the targets. As demonstrated by participants’ directional (signed) errors, both groups gravitated their responses to the center of the referent space, thus, considering the referent space as one spatial entity. This bias was also shown in other spatial scaling studies [79, 80, 82, 83]. Although in this original study from Szubielska et al. [81], scaling factors were manipulated systematically, response times were not reported, which limits conclusions about spatial scaling strategies. Moreover, only one scaling direction (i.e., scaling up) was tested. Consequently, it remains poorly understood, 1) which strategies individuals with blindness adopt for spatial scaling and 2) how blind individuals manage to scale in both directions (up and down).

### 1.4 The aim and hypotheses of the current study

Building upon the theoretical background, we assumed that late blind people can visualize using the “mind’s eye”. Thus, we expected them to use similar mental transformation strategies as sighted people, which would result in response times and errors that are a linear function of different scaling factors (1:3, 1:2, 1:1, 2:1, 3:1). If scaling up and down are processed similarly, result patterns should be V-shaped (best described by a quadratic function) for late blind individuals and blindfolded sighted controls (H1). In contrast, given that early blind people cannot adopt visuo-spatial strategies, they will likely use other ways [3, 4, 6], by adopting relative distances or absolute strategies for spatial scaling. The absolute distance strategy might be linked to using an egocentric frame of reference. When taking the egocentric reference frame, individuals focus on the body’s experiences (e.g., the distance that the hand moves on the map from a chosen edge of the space to the target). Whereas using absolute distance is rather ineffective and error-prone, it is also possible that early blind individuals use relative distances. The relative distance strategy can be linked to verbal coding, as the distance remembered in relative form (e.g., one-fifth from the right) can be verbalized, repeated silently and easily remembered [29]. Verbalizing as a strategy to support spatial performance has been found in several studies with congenital blind people [4, 6]. Following this argumention, response times and errors may not relate to scaling factors in early blind participants (H2).

To answer these hypotheses, we recruited late blind (Experiment 1) and early blind individuals (Experiment 2). For each group, we tested an additional sample of blindfolded sighted adults who were matched to either early and late blind individuals on a number of demographic variables. Two experiments were necessary to investigate the spatial scaling strategies among blind adults who have or do not have visual memories. In each Experiment, we used a methodology that allowed measuring errors and response times, which enabled to differentiate between spatial scaling strategies.

## 2. Experiment 1: Late blind versus blindfolded sighted controls

### 2.1 Materials and methods

#### 2.1.1 Participants

We recruited late blind volunteers from four big cities in Poland. We contacted potential participants via personal contacts, social media, sport clubs and associations of blind people. After having tested the majority of blind participants, we started recruiting sighted participants, who were matched to blind participants in terms of gender, age (± 1 year), handedness, and educational level. These sighted participants were reached out via personal contacts and social media.

The final sample of Experiment 1 comprised 58 participants, amongst which there were 29 late blind adults aged from 18–62 years (*M* = 40.90, *SD* = 12.66, 12 females, 2 left-handed) and 29 sighted adults aged from 19–63 years (*M* = 41.14, *SD* = 12.71, 12 females, 2 left-handed). Sighted participants had normal or corrected-to-normal vision. Each group of blind and sighted participants comprised of people with primary education (*n* = 1), secondary education (*n* = 8), vocational training (*n* = 6), and higher education (*n* = 14). The age of sight loss in the late blind group was at 3 years of age (*n* = 1), 4 (*n* = 1), 5 (*n* = 1), 6 (*n* = 4), 7 (*n* = 1), 9 (*n* = 1), 10 (*n* = 2), 11 (*n* = 2), 12 (*n* = 1), 13 (*n* = 2), 15 (*n* = 1), 16 (*n* = 3), 18 (*n* = 1), 19 (*n* = 1), 26 (*n* = 1), 30 (*n* = 2), 34 (*n* = 1), 35 (*n* = 2) or 39 years (*n* = 1, *M* = 15.90 years, *SD* = 10.69). Importantly, all blind participants reported visual memories and might use visual imagery (e.g., when dreaming). Sixteen late blind participants had currently a sense of light and 13 of them were totally blind. Table 1 presents information about the causes of the sight loss. The majority (72.41%) of the late blind participants used braille alphabet. Most of the blind participants had never (*n* = 8) or rarely (*n* = 12) used tactile graphics. Five of them used tactile graphics sometimes and four often. Similarly, the majority of the sighted participants had never used tactile graphics (*n* = 27), with one person using them rarely and one person using them sometimes. The Ethical Committee of the Institute of Psychology of The John Paul II Catholic University of Lublin approved all procedures performed in the current study involving human participants (reference number KEBN 16/2020). Participants were recruited between May 2021 and August 2022. Before starting the main task, each person signed a written informed consent to participate. All participants provided the signatures and were willing to participate. The institutional ethics committee approved the consent procedure. According to an a priori power analysis [93], comparing samples of late blind and sighted individuals would need a minimum sample size of *N* = 56 (*n* = 28 for each group). These power analyses were based on a moderate effect size of *f* = .30 (based on effect sizes from Szubielska et al. [81]), significance levels of *p* < .05, and a power of .80 to reveal a between-participants effect in a repeated measures ANOVA. Therefore, it seems that the analyses are adequately powered to detect effects.

**Table 1:** Frequency of the causes of the sight loss in the group of late blind participants (*n* = 29).

Cause of the sight loss	N	%
Brain tumor	1	3.4
Cataract	1	3.4
Congenital glaucoma	1	3.4
Disease	5	17.2
Genetic disease	3	10.3
Injury	5	17.2
Optic nerve atrophy	4	13.8
Optic nerve tumor	2	6.9
Retinal degeneration	4	13.8
Retinoblastoma	2	6.9
Toxoplasmosis	1	3.4

#### 2.1.2 Materials

**Fig 1:**
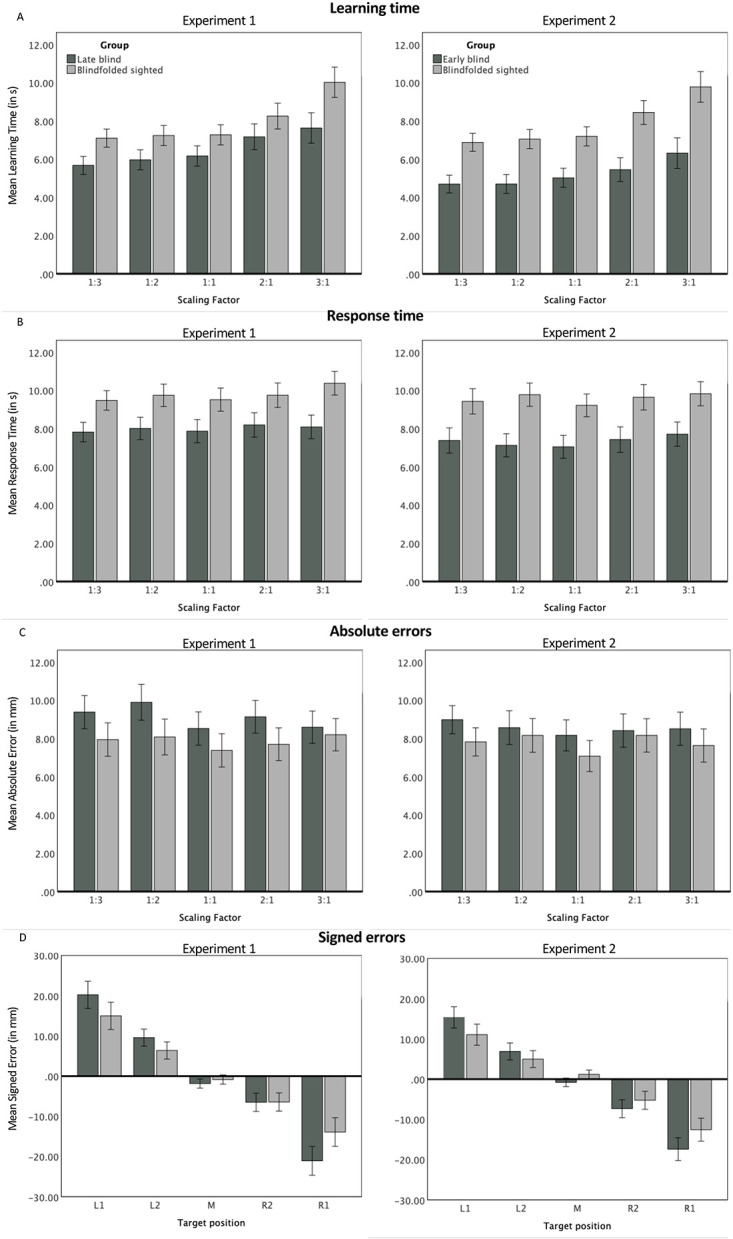
(A) Learning times, (B) response times and (C) absolute errors as a function of scaling factors in both experiments. (D) The effect of target position on signed errors. Dependent variables are presented separately for each visual status group (Experiment 1: late blind participants vs. sighted controls, Experiment 2: early blind participants vs. sighted controls). *Note*. Error bars represent +/- 1 *SE*. Labels on the x axis from panel D mean: L1 = first from left, L2 = second from left, M = middle, R2 = second from right, R1 = first from right. Negative values of signed errors suggest that the disc was placed too far to the left on the referent space; positive values indicate that the disc was placed too far to the right on the referent space.

The spatial scaling task was conducted using 25 embossed graphics. These graphics were made of wooden-like boards (11 x 29 cm). A rectangle map made of black felt was glued onto each wooden-like board. A round target made of smooth material was then glued onto each rectangular map of felt (the same maps were used in a recent study among children; see Fig 1 at [81]). We decided to use tactile graphics produced in such a "collage" technique (textured pictures) because studies on the perception of tactile graphics revealed that graphics produced in raised-line technique can be difficult to interpret for blind and blindfolded sighted participants [94, 95].

Sizes of the maps and the target varied according to five scaling factors (1:3, 1:2, 1:1, 2:1, 3:1). For each scaling factor, there were five equidistant positions of the target: first from left (L1), second from left (L2), middle (M), second from right (R2) and first from right (R1). Target location (5) and scaling factor (5) were combined in a full-factorial design, resulting in a total of 25 maps. In addition to these 25 maps, there was an empty referent space (90 x 30 mm). A round response disc made from the same smooth material as the target (with a diameter of 15 mm) was not attached to the referent space so that the participants could move it freely. The participant’s task was to learn about the target location in the map and to place the response disc at the same location as the target on the referent space. Table 2 shows the dimensions of the maps for each scaling factor as well as the target sizes and coordinates. A digital SLR camera (Canon EOS 600D) was placed on a rack to take pictures of each board on which participants gave a response. We also used two laptops to measure learning and response times (using the stopwatch tool).

**Table 2:** Stimuli sizes and coordinates of the target.

Scaling factor	Target diameter [mm]	Map sizes (dimensions of the black rectangle) [mm]		Coordinates of the target position [mm]		Map number
		OX	OY	OX	OY	
**1:3**	5	30	10	5	5	1
				10	5	2
				15	5	3
				20	5	4
				25	5	5
**1:2**	7.5	45	15	7.5	7.5	6
				15	7.5	7
				22.5	7.5	8
				30	7.5	9
				37.5	7.5	10
**1:1**	15	90	30	15	15	11
				30	15	12
				45	15	13
				60	15	14
				75	15	15
**2:1**	30	180	60	30	30	16
				60	30	17
				90	30	18
				120	30	19
				150	30	20
**3:1**	45	270	90	45	45	21
				90	45	22
				135	45	23
				180	45	24
				225	45	25

#### 2.1.3 Procedure

Participants were tested individually by two experimenters to ensure a smooth procedure. Each participant was seated comfortably at a table. Sighted participants were blindfolded prior to and throughout the study. At the beginning of the scaling task, Experimenter 1 stood to the right of the participant. He sequentially presented the boards with maps to the participant and measured learning times. Experimenter 2, who sat to the left of the participant, was concerned with giving the referent space to the participant, taking pictures of the responses, and measuring the response times. Five practice trials prior to the test phase were run to familiarize the participant with the testing procedure.

Once the experimenters made sure that participants understood the instructions, the main task began. A random sequence of the maps was generated for each person prior to the task and maps were presented in this respective random order. Before each trial, Experimenter 2 read out loud the map number from this list in accordance to the randomized order. Experimenter 1 placed the appropriate map in front of the participant and said “now”. This was the signal for participants to start learning about the target location. Once the participant started exploring the map, Experimenter 1 turned on a stopwatch, measuring the time to learn about the location of the target in the map (learning time). The participant was instructed to explore the map using two hands. As soon as they memorized the target location, they were instructed to say “now” and placed the hands on their lap. At the “now” signal, Experimenter 1 stopped the stopwatch.

Immediately afterwards, Experimenter 2 placed the referent space, with the disc located to the right from this referent space, in front of the participant. Experimenter 2 said “now”, turning on the stopwatch to measure response times. Each participant was instructed to take the response disc and to locate it on the referent space at the same spot as presented in the map. After completing the task, the participant said “now” and put the hands back on the lap. At the “now” signal, Experimenter 2 stopped the stopwatch.

Overall, the participants were presented with 25 maps (trials) within the spatial scaling task as the experiment was designed to include five scaling factors and five target locations. Afterwards, participants also answered questions about demographics and their experience of using tactile graphics. Moreover, blind participants were asked about the aetiology of sight loss and lifelong visual experience. Each participant self-declared the details regarding the visual status and other information.

#### 2.1.4 Dependent variables and data preparation

In this experiment, four dependent variables were measured: learning times, response times, absolute errors, and signed errors.

**Learning times.** Learning times were measured from the moment when participants started exploring the map until participants stopped the map exploration and said “now”.

**Response times.** Response times were measured from the moment when the experimenter said “now” to signal the start of the localization procedure until participants completed the task and said “now”.

**Absolute errors.** To measure the accuracy of the spatial scaling performance, we used an algorithm programmed for the current study in open-source software (using Python programming language). The program analyzed the pictures and provided the x- and y-coordinates of each participant’s answer. Next, we computed the Euclidean distance between a participant’s response and the correct target location. It was found that participants produced some reversal errors and thus, gave responses on the opposite side of the referent space when compared to the target’s location on the map. These reversal errors occurred in 20.75% of all cases, but blind participants and sighted controls did not differ regarding the number of reversal errors (see S1 Table). Given that these reversal errors produced large variability in the data, we used a corrected version of these absolute errors similar to coding approaches in previous research (e.g., [77]). According to this approach, we coded these reversal errors as if the target was located on the correct side on the horizontal dimension; however, as can be seen in the results section, findings did not differ when using the uncorrected version of these absolute errors.

**Signed errors.** Signed errors reflect the direction of the errors and thus, inform us about potential underlying biases (e.g., whether responses gravitate towards the midpoint or the borders of a space). To compute signed errors, we subtracted the *x*-coordinate of the respective target location from the *x*-coordinate of each participant’s answer (in mm; for similar procedures, see e.g., [56, 79]). These values were then averaged for each position on the horizontal dimension (L1, L2, M, R2, R1). Negative values of signed errors suggest that the disc was placed too far to the left on the referent space; positive values indicate that the disc was placed too far to the right on the referent space.

We identified and deleted outliers (*M* ± 3 *SD*s) in participants’ learning times, response times, absolute errors and signed errors. Outliers comprised 2.07% of all cases (from a total of 1450 responses) with respect to learning times, 2.07% of response times, 0.55% of absolute errors and 0.24% of signed errors.

#### 2.1.5 Statistical approach

We conducted a 5 x 2 mixed analysis of variance (ANOVA) with scaling factor (1:3, 1:2, 1:1, 2:1, 3:1) as a within-participant variable and visual status (late blinds vs. blindfolded sighted controls) as a between-participants factor for the following dependent variables: learning times, response times, and absolute errors. For the ANOVA using signed errors, we additionally included a within-participant factor of target position (L1, L2, M, R2, R1). Prior to computing these mixed ANOVAs, we tested the assumptions. The between-groups variances were homogenous. In the case of a lack of sphericity, we used a Greenhouse–Geisser correction.

Due to much missing data from one late blind participant (as a result of identifying outliers), she and her sighted matched person were not included in the analyses using learning and response times. Thus, analyses using learning and response times comprised only 28 pairs of participants (*n* = 56). By contrast, the analysis using absolute errors comprised the whole sample (*n* = 58). Due to the inclusion of the additional within-participant factor in the analysis using signed errors, missing data resulted in more rejected participants. Thus, the analysis using signed errors included 25 pairs of participants (*n* = 50). The datasets for both experiments are available in the Figshare repository under this link: dx.doi.org/10.6084/m9.figshare.23567415.

### 2.2 Results

During childhood, people learn to navigate in space. To ensure that participants who lost sight in early childhood (between the ages of three and five) did not produce any statistical bias, we first conducted each analysis with a reduced sample by excluding three participants who lost vision in early childhood. Analyses revealed similar results. Thus, in the following sections, we report the results of the statistical analyses based on the whole sample.

#### 2.2.1 Learning times

The ANOVA revealed a significant main effect of scaling factor (see Table 3, for descriptive statistics; see Table 4, for inferential statistics), which was best explained by a linear function, *F*(1, 54) = 58.32, *p* < .001, η_p_^2^ = .52, suggesting that participants needed longer to explore larger maps (see Fig 1, panel A left). A marginally significant main effect of the visual status (*p* = .074) showed that late blind participants tended to learn faster in comparison to their sighted counterparts. The interaction effect did not reach significance (*p* > .097).

**Table 3:** Descriptive statistics (means and standard deviations) for the learning times (in s), response times (in s), and absolute errors (in mm) as a function of scaling factors in late blind and blindfolded sighted participants (Experiment 1).

		Scaling factor									
		1:3		1:2		1:1		2:1		3:1	
		*M*	*SD*	*M*	*SD*	*M*	*SD*	*M*	*SD*	*M*	*SD*
**LTs** ^a^	**Late blind**	5.67	2.04	5.96	2.49	6.17	2.69	7.17	3.17	7.63	3.38
	**Blindfolded sighted**	7.10	2.91	7.24	3.06	7.27	2.91	8.25	3.94	10.02	4.89
	**All participants**	**6.38**	**2.59**	**6.60**	**2.83**	**6.72**	**2.83**	**7.71**	**3.59**	**8.83**	**4.34**
**RTs**	**Late blind**	7.83	2.39	8.02	2.87	7.87	3.04	8.20	2.95	8.10	2.90
	**Blindfolded sighted**	9.48	3.00	9.75	3.30	9.52	3.37	9.76	3.77	10.38	3.62
	**All participants**	**8.65**	**2.81**	**8.88**	**3.19**	**8.70**	**3.29**	**8.98**	**3.45**	**9.24**	**3.45**
**AEs**	**Late blind**	9.39	4.67	9.90	5.40	8.53	5.05	9.14	4.52	8.60	4.57
	**Blindfolded sighted**	7.95	4.72	8.09	4.68	7.39	4.32	7.70	4.72	8.20	4.52
	**All participants**	**8.67**	**4.71**	**8.99**	**5.09**	**7.96**	**4.69**	**8.42**	**4.64**	**8.40**	**4.51**

^a^Note. LTs = learning times, RTs = response times, AEs = absolute errors.

**Table 4:** Inferential statistics with learning times, response times, and absolute errors in Experiment 1. Significant effects are marked in bold.

	Learning times				Responses times				Absolute errors			
	*Dfs*	*F*	*p*	η_p_^2^	*Dfs*	*F*	*p*	η_p_^2^	*dfs*	*F*	*p*	η_p_^2^
Scaling factor	**2.92, 157.66**	**30.43**	**< .001**	**.36**	**4, 54**	**3.02**	**.019**	**.05**	3.05, 170.80	1.15	.335	.02
Visual status	1, 54	3.31	.074	.06	**1, 54**	**4.88**	**.031**	**.08**	1, 56	1.37	.247	.02
Scaling factor x Visual status	2.92, 157.66	2.16	.097	.04	4, 54	1.16	.331	.02	3.05, 170.80	.56	.646	.01

#### 2.2.2 Response times

The analysis using response times showed significant main effects of scaling factor and visual status (see Table 3, for descriptive statistics; see Table 4, for inferential statistics). The effect of scaling factor was best explained by a linear function, *F*(1, 54) = 8.58, *p* = .005, η_p_^2^ = .14. Response times increased linearly with larger size of the previously encoded map (see Fig 1, panel B left). The between-participant comparison revealed that late blind participants responded faster (*M*_late blind_ = 8.00, *SD*_late blind_ = 0.26) than blindfolded sighted controls (*M*_blindfolded sighted_ = 9.78, *SD*_blindfolded sighted_ = 0.30). The interaction effect was nonsignificant (*p* > .33).

#### 2.2.3 Absolute errors

There were no significant effects in the analysis using absolute errors (all *p*s > .33; see Table 3, for descriptive statistics; see Table 4, for inferential statistics; see Fig 1, panel C left). Analogous, nonsignificant results were also yielded in the analysis conducted on uncorrected absolute errors (for *n* = 58). Both types of analyses with corrected and uncorrected absolute errors as dependent variables provided null effects when conducted with the reduced sample (*n* = 56) that was used in the analyses with learning and response times.

#### 2.2.4 Signed errors

The only significant result was an effect of target position (see Table 5), which was best explained by a linear function, *F*(1, 48) = 49.87, *p* < .001, η_p_^2^ = .51. Signed errors were larger for the left-most as well as for the right-most target locations in comparison to the central location. Fig 1 (panel D left) shows a noticeable tendency for a response shift towards the center of the referent space which can be seen for both visual status groups.

**Table 5:** Inferential statistics with signed errors in Experiment 1. Significant effects are marked in bold.

	Signed errors			
	*Dfs*	*F*	*p*	η_p_^2^
Target position	**1.43, 68.71**	**41.62**	**< .001**	**.46**
Scaling factor	4, 192	0.34	.850	.01
Visual status	1, 48	0.01	.933	.00
Target position x Scaling factor	10.13, 486.07	1.03	.415	.02
Target position x Visual status	1.43, 68.71	1.29	.274	.03
Target position x Scaling factor x Visual status	10.13, 486.07	1.02	.428	.02

### 2.3 Discussion

In accordance to previous studies in the haptic domains [83], we found that participants of both visual status groups needed longer to learn about the maps when these were larger. Moreover, our findings showed that learning times did not differ between the visual status groups. In contrast, late blind individuals responded faster than sighted participants as indicated by their higher response times. These faster responses probably exist to their higher haptic experience which is in line with several previous studies suggesting that blind adults are faster in haptic spatial tasks than sighted adults (see e.g., [5, 13, 96, 97]). With respect to absolute or signed errors, there were no differences between groups. Both groups gravitated their responses to the middle of the referent space.

Predictions regarding the usage of mental transformation strategies among late blind and blindfolded sighted participants in the haptic domain were not confirmed. The influence of scaling factor on absolute errors was insignificant, irrespective of the visual status. Moreover, although scaling factor influenced response times, the relation between scaling factor and response times was best explained by a linear function and not by a V-shaped function (as predicted). Similar to participants’ learning times, participants needed longer to respond after learning about a larger map (3:1) as compared to a smaller map (1:3). From this pattern of results, it hard to pinpoint the exact spatial scaling strategy. In contrast to previous studies [80, 81, 83], the present results did not yield evidence for mental transformation strategies in neither visual status group. However, our results are in line with a number of previous studies demonstrating that sighted and late blind adults respond highly similar [3, 6].

## 3. Experiment 2: Early blind *versus* blindfolded sighted controls

### 3.1 Materials and methods

The way to recruit participants was similar to Experiment 1. In addition, in this study, we used the same materials and followed the same experimental procedure. Data preparations and statistical approaches were analogous as in Experiment 1.

#### 3.1.1 Participants

The sample of Experiment 2 comprised 66 participants, amongst which there were 33 early blind adults aged from 21–61 years (*M* = 36.15, *SD* = 12.57, 13 females, 2 left-handed) and 33 sighted adults aged from 21–61 years (*M* = 36.30, *SD* = 12.44, 13 females, 2 left-handed). Sighted participants had normal or corrected-to-normal vision. Three blind people were not totally blind at birth, but lost sight before turning three years. As they declared having no visual memories, we qualified them as early blind.

In each group of participants, there were people with secondary education (*n* = 14), vocational training (*n* = 1) and higher education (*n* = 18). Seventeen early blind participants had a sense of light and sixteen were totally blind. Table 6 presents information about the causes of the sight loss. Almost all (*n* = 32, 97%) early blind participants used braille alphabet. Two blind participants have never used tactile graphics. Sixteen of them used tactile graphics rarely, six sometimes, seven often and two very often. Similarly, most of the sighted participants had never used tactile graphics (*n* = 29) and four people used them rarely.

**Table 6:** Frequency of the causes of the sight loss in the early blind group (*n* = 33).

Cause of the sight loss	N	%
Birth injury	1	3
Congenital defect	5	15.2
Eyeball underdevelopment	1	3
Genetic disease	1	3
Optic nerve atrophy	2	6.1
Optic nerve hypoplasia	3	9.1
Retinal degeneration	2	6.1
Retinoblastoma	3	9.1
Retinopathy of prematurity	12	36.4
Toxoplasmosis	1	3
Unknown	2	6.1

#### 3.1.2 Outliers and reversal errors

Outliers (*M* ± 3 *SD*s) comprised 1.64% of all cases (from a total of 1650 responses) with respect to learning times, 2.18% of response times, 0.48% of absolute errors and 0.24% of signed errors. Due to many missing responses times (as a result of identifying outliers) in one early blind participant, she and her sighted matched person were both excluded from the analysis of variance. Thus, the ANOVA on response times was conducted with *n* = 64 participants. For the same reason, the analysis on the signed errors was conducted for 31 pairs (*n* = 62). For all the other dependent variables (i.e., learning times, absolute errors), the whole sample (*N* = 66) was included.

In analogy to Experiment 1, we checked data for reversal errors. Reversal errors occurred for 20.58% of all answers in the present experiment. As in Experiment 1, the number of reversal errors did not differ between early blind and sighted participants (see S1 Table). Again, we coded these reversal errors as if the target was located on the correct side on the horizontal dimension; however, results did not differ when using the uncorrected version of these absolute errors.

### 3.2 Results

To ensure that participants who were not totally blind since birth but lost sight before turning three years did not produce any statistical bias, we first conducted each analysis with a reduced sample by excluding these three participants. Analyses revealed similar results. Thus, in the following sections, we report results of the statistical analyses based on the whole sample (*N* = 66; 33 pairs of participants).

#### 3.2.1 Learning times

The ANOVA revealed a significant main effect of scaling factor (see Table 7, for descriptive statistics; see Table 8, for inferential statistics). This effect was best explained by a linear function, *F*(1, 64) = 56.76, *p* < .001, η_p_^2^ = .47. In line with Experiment 1, participants needed longer to explore larger maps. In addition, the main effect of visual status was significant. Blind participants explored the maps faster (*M*_early blind_ = 5.51, *SD*_early blind_ = 3.42) than their sighted controls (*M*_blindfolded sighted_ = 7.85, *SD*_blindfolded sighted_ = 3.40). Given that the interaction of scaling factor by visual status was also significant, we conducted two one-way ANOVAs, separately for each group, with scaling factor as a within-participant variable. Both analyses revealed a significant main effect of scaling factor, for early blind, *F*(1.99, 63.83) = 10.27 *p* < .001, η_p_^2^ = .24; for blindfolded sighted controls, *F*(2.37, 75.83) = 28.11 *p* < .001, η_p_^2^ = .47. Again, it was found that linear functions described the pattern of results for early blind, *F*(1, 32) = 15.35 *p* < .001, η_p_^2^ = .32; for blindfolded sighted controls, *F*(1,32) = 44.53 *p* < .001, η_p_^2^ = .58. Given the significant interaction effect, we checked pairwise comparisons for each scaling factor, which were all significant (all *p*s < .01). In each scaling factor condition, early blind participants encoded maps faster than blindfolded sighted participants. However, a look at Fig 1 (panel A right) reveals that this difference was especially pronounced for the largest maps (3:1). Analogous analyses conducted with the reduced sample (*n* = 64) based on the outlier analysis for response times yielded the same pattern of results.

**Table 7:** Descriptive statistics (means and standard deviations) for the learning times (in s), response times (in s), and absolute errors (in mm) as a function of scaling factors in early blind and blindfolded sighted participants (Experiment 2).

		Scaling factor									
		1:3		1:2		1:1		2:1		3:1	
		*M*	*SD*	*M*	*SD*	*M*	*SD*	*M*	*SD*	*M*	*SD*
**LTs** ^a^	**Early blind**	4.97	2.88	4.89	2.67	5.37	3.42	5.76	3.69	6.55	4.47
	**Blindfolded sighted**	6.85	2.79	7.03	3.08	7.19	2.78	8.41	3.71	9.77	4.65
	**All participants**	**5.91**	**2.97**	**5.96**	**3.05**	**6.28**	**3.23**	**7.08**	**3.91**	**8.16**	**4.80**
**RTs**	**Early blind**	7.39	3.77	7.13	3.18	7.05	3.07	7.43	4.06	7.72	3.41
	**Blindfolded sighted**	9.43	3.71	9.78	3.68	9.22	3.65	9.65	3.45	9.83	3.73
	**All participants**	**8.41**	**3.85**	**8.46**	**3.66**	**8.14**	**3.52**	**8.54**	**3.90**	**8.77**	**3.70**
**AEs**	**Early blind**	8.99	4.18	8.58	4.62	8.17	4.82	8.42	4.87	8.52	5.12
	**Blindfolded sighted**	7.83	4.29	8.17	5.49	7.09	4.50	8.17	5.14	7.64	4.85
	**All participants**	**8.41**	**4.24**	**8.37**	**5.04**	**7.63**	**4.66**	**8.29**	**4.97**	**8.08**	**4.97**

^a^Note. LTs = learning times, RTs = response times, AEs = absolute errors.

**Table 8:** Inferential statistics with learning times, response times, and absolute errors in Experiment 2. Significant effects are marked in bold.

	Learning times				Responses times				Absolute errors			
	*Dfs*	*F*	*p*	η_p_^2^	*dfs*	*F*	*p*	η_p_^2^	*dfs*	*F*	*p*	η_p_^2^
Scaling factor	**2.28, 146.07**	**36.56**	**< .001**	**.36**	**3.45, 213.68**	**3.11**	**.021**	**.05**	3.42, 219.20	1.05	.384	.02
Visual status	**1, 64**	**8.37**	**.005**	**.12**	**1, 62**	**6.70**	**.012**	**.10**	1, 56	1.37	.247	.01
Scaling factor x Visual status	**2.28, 146.07**	**3.50**	**.027**	**.05**	3.45, 213.68	.84	.498	.01	3.42, 219.20	.53	.469	.01

#### 3.2.2 Response times

The analysis on response times showed that both main effects of scaling factor and visual status were significant (see Table 7, for descriptive statistics; see Table 8, for inferential statistics). Early blind participants performed the task faster (*M*_early blind_ = 7.34, *SD*_early blind_ = 3.50) than sighted controls (*M*_blindfolded sighted_ = 9.58, *SD*_blindfolded sighted_ = 3.64). The significant effect of scaling factor was best explained by a quadratic function, *F*(1, 62) = 4.74, *p* = .033, η_p_^2^ = .07. As can be seen in Fig 1 (panel B right), the increase in response times with higher scaling factor is particularly evident when scaling down. The interaction was not significant.

As we predicted the relationship between scaling factor and response times to be V-shaped (being best described by a quadratic function) among sighted but not early blind participants, we conducted two additional, separate ANOVAs for early blind (*n* = 32) and sighted participants (*n* = 32). The effect of scaling factor did not reach significance among early blind participants, *F*(2.99, 92.80) = 1.71, *p* = .171, η_p_^2^ = .05. In the blindfolded sighted controls, the effect of scaling factor was close to significance, *F*(4, 124) = 2.40, *p* = .054, η_p_^2^ = .07, and was best explained by a quadratic function *F*(1, 62) = 4.74, *p* = .033, η_p_^2^ = .07.

#### 3.2.3 Absolute errors

There were no significant effects in the analysis with absolute errors (all *p*s > .24; see Table 8). Neither an analysis with uncorrected absolute errors nor with the reduced sample (based on the outlier analysis with response times) yielded different results.

#### 3.2.4 Signed errors

Similar to the results obtained in Experiment 1, there was a significant main effect of target position (see Table 9), which was best explained by a linear function, *F*(1, 48) = 45.85, *p* < .001, η_p_^2^ = .43. As can be seen in Fig 1 (panel D right), participants showed a response bias towards the center of the space.

**Table 9:** Inferential statistics with the signed errors in Experiment 2. Significant effects are marked in bold.

	Signed errors			
	*dfs*	*F*	*p*	η_p_^2^
Target position	**1.39, 83.34**	**37.63**	**< .001**	**.38**
Scaling factor	3.44, 206.36	.85	.483	.01
Visual status	1,60	1.57	.215	.03
Target position x Scaling factor	10.39, 623.32	1.44	.154	.02
Target position x Visual status	1.39, 83.34	1.04	.334	.02
Target position x Scaling factor x Visual status	10.39, 623.32	1.01	.434	.02

## 4. Comparing late and early blind participants

We additionally compared spatial scaling between late and early blind people by conducting a separate set of repeated measures ANOVAs. The design was the same as in the previously reported analyses apart from the between-participants factor, which now comprised late vs. early blind participants.

### 4.1 Results

#### 4.1.1 Learning times

Consistently with the results of the previous analyses on learning times, there was a significant main effect of scaling factor (see Table 10, for inferential statistics), which was best explained by linear function, *F*(2.92, 157,65) = 30.43, *p* < .001, η_p_^2^ = .36. Early and late blind participants needed longer to explore larger maps. No other effect was significant.

**Table 10:** Inferential statistics with learning times, response times, and absolute errors for late and early blind participants. Significant effects are marked in bold.

	Learning times (*n*_EB_^a^ = 33, *n*_LB_ = 28)				Responses times (*n*_EB_ = 32, *n*_LB_ = 28)				Absolute errors (*n*_EB_ = 33, *n*_LB_ = 29)			
	*Dfs*	*F*	*p*	η_p_^2^	*dfs*	*F*	*p*	η_p_^2^	*dfs*	*F*	*p*	η_p_^2^
Scaling factor	**2.59, 152.92**	**21.24**	**< .001**	**.26**	4, 233	1.68	.154	.03	3.47, 208.20	1.27	.287	.02
Visual status	1, 59	1.75	.191	.03	1, 58	.68	.412	.01	1, 60	.30	.586	.00
Scaling factor x Visual status	2.59, 152.92	.73	.516	.01	4, 232	.68	.608	.01	3.47, 208.20	.48	.727	.01

^a^Note. EB = early blind; LB = late blind.

#### 4.1.2 Response times

There were no significant effects for the ANOVA with response times (see Table 10).

#### 4.1.3 Absolute errors

There were no significant effects for the ANOVA with absolute errors (see Table 10).

#### 4.1.4 Signed errors

Conforming to the effects found in previous analyses, the main effect of target position was significant (see Table 11) and was qualified by a linear function *F*(1, 60) = 45.85, *p* < .001, η_p_^2^ = .43. No other effects were significant.

**Table 11:** Inferential statistics with signed errors for late and early blind participants. Significant effects are marked in bold.

	Signed errors (*n*_EB_^a^ = 31, *n*_LB_ = 27)			
	*dfs*	*F*	*p*	η_p_^2^
Target position	**1.48, 82.89**	**55.86**	**< .001**	**.38**
Scaling factor	4, 224	.23	.923	.00
Visual status	1, 56	2.10	.153	.03
Target position x Scaling factor	11.16, 624.86	1.00	.442	.02
Target position x Visual status	1.48, 82.89	.72	.450	.01
Target position x Scaling factor x Visual status	11.16, 624.86	.92	.517	.02

^a^Note. EB = early blind; LB = late blind

### 4.2 Discussion

When comparing performance between early blind and blindfolded sighted participants, it was found that early blind participants performed the scaling task quicker than blindfolded sighted participants, as indicated by shorter learning and response times. This result echoes our findings of Experiment 1 and is in line with previous research [5, 13, 96, 97]. Despite this difference, early blind and blindfolded sighted participants did not differ with respect to their absolute errors and directional (signed) errors. That is, both groups tended to gravitate their responses to the midpoint of the space.

When investigating effects of scaling factors, the obtained findings suggest that, early blind participants seemed to use a relative distance strategy for spatial scaling in line with our hypothesis. This interpretation was based on the insignificant effect of scaling factor on either response times nor absolute errors [65, 66]. However, as in Experiment 1, the hypothesis regarding the adoption of mental transformation strategies by blindfolded sighted participants was not fully supported. Although response times of sighted participants suggested the usage of mental transformation strategies as indicated by a trend for a V-shaped relationship between scaling factors and response times, the effect of scaling factor on absolute errors did not reach significance which limits the inference of mental transformation strategies. This non-conclusive pattern of findings may result from the blindfolded sighted people trying to decrease or increase the size of the memorized map in their mental representation while, at the same time, having difficulty to switch from an egocentric perspective to an allocentric perspective. Whereas the haptic perception of the map may have provoked an egocentric perspective, it is a preferrable precondition to switch to an allocentric perspective to accurately perform the task with different scaling factors. Similar difficulties were found among blindfolded sighted children [98].

When comparing performance between late and early blind participants, our findings suggest remarkable similarities in spatial scaling ability between these two groups. These similarities were found for the speed of the scaling process as well as participants’ precision in locating the targets. At the same time, both groups of blind participants are similarly susceptible to a response bias of gravitating responses to the center of the spatial layout. However, it needs to be noted that results of these comparisons should be interpreted with caution as early and late blind participants were not matched in terms of sociodemographic variables. That is, the sample of early blind participants included fewer participants with higher educational attainment than the late blind sample, which limits the comparability between these groups.

## 5. General discussion

The main objective of the current study was to compare the precision and timing of spatial scaling in early blind, late blind adults and blindfolded sighted controls with the goal to explore spatial scaling strategies. We predicted that those participants who may rely on visual imagery (i.e., sighted and late blind) would use mental transformation strategies and that early blind people may use absolute or relative distance strategies. In the case of blindfolded sighted, as well as the late blind participants, result patterns did not lend full support to any of the spatial scaling strategies described in the literature. Thus, the hypothesis that these participants would adopt mental transformation strategies was not supported by the present data. Thus, findings of previous studies were not replicated [80, 81, 83, 84]. Even though in both experiments, a look at the descriptive data for the response times in scaling down conditions might suggest the usage of mental transformation strategies by late blind (Experiment 1) and blindfolded sighted participants (Experiment 1 and Experiment 2), the lack of significant effects on absolute errors prevents considering such an interpretation.

At the same time, the hypothesis of adopting a relative distance strategy by early blind participants was supported. When encoding a map, participants may have focused on the internal relative relationships within the elements, and not on its size per se. Similar conclusions were drawn in studies about recognizing the shape of figures of different sizes presented on tactile graphics [25]. It turned out that in the case of congenitally blind adults (as opposed to sighted controls), the time needed to recognize the shape of a zoomed-up figure did not increase. Overall, it seems that adults who use the “mind’s hand” to carry imagery processes use different spatial scaling strategies than those who can use visual imagery.

The present study revealed interesting similarities and some differences in spatial scaling ability depending on the visual status of participants. The similarities referred to the prone bias of gravitating responses to the center of the board. Regardless of the visual status, adults were susceptible to a response bias and gravitated responses to the center. A similar bias was found in previous studies of spatial scaling in the haptic domain among blindfolded sighted adults [80, 82, 83] and congenitally blind participants [81]. This bias suggests that participants considered the map as one spatial entity [67]. Interestingly, the possibility of visualizing the haptically encoded map did not increase a fine-grained spatial encoding in sighted and late blind participants.

Another similarity across the visual status groups referred to the level of absolute errors. Accuracy did not differ between early blind, late blind, and blindfolded sighted participants. An explanation may refer to the construction of the maps used in the present task. The maps were on purpose held simple and did not contain detailed elements. Considering that late blind and sighted people may have used the “mind’s eye” which is described as having a higher resolution than the “mind’s hand” [16, 34], using more complex maps may result in differences between the visual status groups (but see [99, 100]). Another explanation may be the adoption of different spatial scaling strategies by early blind participants as compared to sighted and late blind participants which may have helped to respond as accurately in the present task. Similar conclusions (i.e., using different strategies depending on the availability of visual imagery) have been drawn from previous research on mental imagery [3–6, 16].

Another interesting conclusion from our study is that the scaling direction (up vs. down) did not influence scaling accuracy among blind and sighted adults in the haptic domain. This result is in line with recent scaling studies in the visual domain [66, 77] (but see [101]). Our findings may have important practical implications for blind people in the context of learning with educational aids such as maps or models. It seems that blind adults may benefit just as effectively from scaled-up models (e.g., representing the construction of a cell) and scaled-down ones (e.g., representing the structure of the Milan Cathedral).

Another similarity referred to the finding that participants with different visual statuses did not differ regarding the number of produced reversal errors. Hence, results of the present study contradict findings of a previous study among adults and children [81]. In that respective study, congenitally blind participants produced reversal errors more frequently than blindfolded sighted controls. These contradicting findings might be explained by the different age groups involved in these studies. Whereas this original study from Szubielska et al. [81] included blind participants aged 8–45 years, the present study included only adult participants. Therefore, the findings from the original study from Szubielska et al. [81] may suggest that encoding the target in the correct horizontal direction might be especially challenging for blind children.

The only measures for which we have found differences between visual status groups concerned the learning and response times. Both groups of blind participants tended to learn the map faster than blindfolded sighted controls, and response times were also faster for blind participants than for blindfolded sighted participants. This pattern of results confirms previous studies in which blind people responded faster than sighted people in spatial tasks in the haptic domain (e.g., [5, 13, 17, 25, 96, 97], but see [9, 59]). Likely, this higher speed in learning and responding goes back to the extensive haptic experience in blind participants.

### 5.1 Limitations and directions for further studies

The present study has several limitations. First, we did not collect participants’ verbal statements about the strategies used (e.g., whether they reported to visualize maps or verbalized information about the target’s location on the map). Access to this information would have allowed us to interpret the results more accurately and may have helped pinpointing the spatial scaling strategies. Second, a few sighted (and possibly also late blind) people in the population experience aphantasia which is defined as the lack of visual imagery. These individuals may retain accuracy in cognitive tasks but adopt different strategies than individuals who can use visual imagery [102]. Although the experience of aphantasia is very rare, it would have been worth asking the sighted and late blind participants whether they experienced visual imagery on a daily basis to ensure that we included participants who can use visual representations. Further limitations relate to the maps used in the study. Their sizes allowed estimating only two scaling factors when scaling up and down. In addition, the maps were very simple with targets being distributed on the horizontal dimension only, and without landmarks. Maps used in real-life situations are naturally more complex and future studies may include such complex maps reflecting higher ecological validity. Last but not least, we only assessed spatial scaling abilities in participants’ peripersonal space (i.e., the area close to the participant’s body which was possible to reach with their hands without walking). In natural settings, we primarily use maps to navigate and walk in large-scaled spaces. Thus, it is unclear whether the present findings can be generalized to these large-scale situations.

Further studies should overcome the abovementioned limitations. Additionally, follow-up research on spatial scaling could investigate spatial scaling ability in blind and sighted participants of different ages. Furthermore, it would be important to investigate spatial scaling ability in older adults given that in this developmental period, individuals sometimes become visually impaired.

### 5.2 Conclusions

In the current study, we have shown for the first time that adults with blindness (regardless of their visual experience) can scale simple tactile maps faster and as accurately as blindfolded sighted individuals. Moreover, our study suggests that early blind adults seem to adopt a relative distance scaling strategy. In contrast, participants who can rely on visual imagery seem to use other spatial scaling strategies, which unfortunately cannot be identified given the unconclusive result pattern in the present study. Overall, it seems that a lack of visual imagery does not impair early blind adults’ spatial scaling ability but causes them to use a different strategy than sighted and late blind individuals.

## Supporting information

S1 Table Inferential statistics with reversal errors in Experiment 1 and 2. (DOCX)
