# Supplementary material for: Adults’ spatial scaling of tactile maps: Insights from studying sighted, early and late blind individuals
Source: PLoS One. 2024 May 30;19(5):e0304008. doi: 10.1371/journal.pone.0304008 (PMC11139347; doi:10.1371/journal.pone.0304008)
Supplement: S1 Table — (DOCX) [file pone.0304008.s001.docx]

**Supporting information**

To verify whether visual status or scaling factor affected the number of reversal errors, we performed two additional ANOVAs, one for each experiment, with the number of reversal errors as the dependent variable, scaling factor as a within-subject factor, and visual status as a between-subject factor. All factors in both analyses provided null effects (see S1 Table).

**S1 Table. Inferential statistics with reversal errors in Experiment 1 and 2.**

|  | **Experiment 1**  #Reversal errors | | | | **Experiment 2**  #Reversal errors | | | |
| --- | --- | --- | --- | --- | --- | --- | --- | --- |
|  | ***dfs*** | ***F*** | ***p*** | **η_p_^2^** | ***dfs*** | ***F*** | ***p*** | **η_p_^2^** |
| Scaling factor | 4, 224 | .89 | .470 | .02 | 3.46, 221.69 | .67 | .590 | .01 |
| Visual status | 1, 56 | .52 | .475 | .01 | 1, 64 | .86 | .358 | .01 |
| Scaling factor x Visual status | 4, 224 | .427 | .789 | .01 | 3.46, 221.69 | .83 | .490 | .01 |
